# Antiplatelet therapy and the outcome of subjects with intracranial injury: the Italian SIMEU study

**DOI:** 10.1186/cc12575

**Published:** 2013-03-21

**Authors:** Andrea Fabbri, Franco Servadei, Giulio Marchesini, Carolina Bronzoni, Danilo Montesi, Luca Arietta

**Affiliations:** 1 Dipartimento Emergenza, Presidio Ospedaliero Morgagni-Pierantoni, Azienda Unità Sanitaria Locale di Forlì, via Forlanini 34, 40121, Forlì, Italy; 2 Dipartimento Emergenza-Urgenza, Struttura Complessa di Neurochirurgia - Neurotraumatologia, Azienda Ospedaliera Universitaria di Parma, viale Gramsci 14, 43126, Parma, Italy; 3 Dipartimento di Scienze Mediche e Chirurgiche, Università di Bologna, via Massarenti 9, 40138, Bologna, Italy; 4 Dipartimento di Informatica, Scienza e Ingegneria, Università di Bologna, Via Mura Anteo Zamboni 7, 40127, Bologna, Italy

## Abstract

**Introduction:**

Pre-injury antithrombotic therapy might influence the outcome of subjects with head injuries and positive computed tomography (CT) scans. We aimed to determine the potential risk of pre-injury antiplatelet drug use on short- and long-term outcome of head injured subjects admitted to emergency departments (EDs) in Italy for extended observation.

**Methods:**

A total of 1,558 adult subjects with mild, moderate and severe head injury admitted to Italian EDs were studied. In multivariable logistic regression analyses, the short-term outcome was assessed by an evaluation of head CT scan at 6 to 24 hours after trauma and the long-term outcome by the Glasgow outcome scale (GOS) at six months.

**Results:**

Head CT scan comparisons showed that 201 subjects (12.9%) worsened. The risk of worsening was increased two fold by the use of antiplatelet drugs (106, 19.7% treated versus 95, 9.3% untreated; relative risk (RR) 2.09, 95% CI 1.63 to 2.71). The risk was particularly high in subjects on clopidogrel (RR 5.76, 95% CI 3.88 to 8.54), independent of the association with aspirin. By logistic regression, 5 of 14 items were independently associated with worsening (Glasgow coma scale (GCS), Marshall category, antiplatelet therapy, intraventricular hemorrhage, number of lesions). After six months, only 4 of 14 items were predictors of unfavorable outcome (GOS 1 to 3) (GCS score, Marshall category, age in decades, intracerebral hemorrhage/contusion). The risk increased by 50% in the group treated with antiplatelet therapy (RR 1.58, 95% CI 1.28 to 1.95; *P *< 0.001).

**Conclusions:**

Antithrombotic therapy (in particular clopidogrel) is a risk factor for both short-term and long-term unfavorable outcome in subjects with head injury, increasing the risk of progression and death, permanent vegetative state and severe disability.

## Introduction

Subjects admitted to the emergency department (ED) with intracranial lesions following head injury are a special challenge for emergency physicians. They represent a heterogeneous group of patients with large variability as to injury severity, clinical course, neurological recovery and overall outcome [1].

Worsening detected by imaging and clinical deterioration are associated with an unfavorable outcome, and a group of predictor variables has been related to worsening-type lesions and future events [2-4]. In a few cases, progression is extremely rapid and the ultimate outcome might be unfavorable because of delayed transfer to neurosurgical units; in other cases the lesions do not progress and the final outcome is usually favorable.

In the last decade the use of antithrombotic therapy with antiplatelet drugs has grown considerably, as an effect of national and international guidelines promoting their widespread use to prevent cardiovascular events in high-risk populations and particularly in older people [5,6]. In the same period, the epidemiology of the trauma population has also changed, with a larger and larger prevalence of older age-groups [7], where antiplatelet drug use is more prevalent, in the presence of comorbidities [8,9].

The aim of this study was to test the effect of pre-injury antiplatelet therapy on short- and long-term outcomes in subjects with head injury and a positive computed tomography (CT) scan at first evaluation.

## Methods

### Study design and settings

This multicenter observational study included all adult subjects, who attended 32 Italian EDs of community and regional hospitals for mild, moderate or severe head injury and intracranial lesions within 24 hours from the event (from January to December 2009). The participating centers represented a wide variety of facilities, distributed across the country, to increase external validity and to make the results generalizable to the majority of subjects observed for head injury. The centers included hospitals with neurosurgical units, hospitals with teleradiology-consulting systems connected with a neurosurgical center and hospitals without neurosurgical and teleradiology facilities.

Adult subjects ≥ 18 years old with mild (Glasgow coma scale (GCS) = 15 to 14) or moderate to severe (GCS ≤ 13) head injury within 24 hours of trauma and a positive head CT scan at their first evaluation in the ED were included in the study. The subjects were all consecutive patients with a positive head CT scan without indication of urgent (within 7 days) neurosurgical hematoma/hemorrhage evacuation (Marshall category 2 to 4 at entry).

Excluded were subjects in the presence of: a) an initial head CT scan requiring urgent neurosurgical intervention (Marshall category 5) or not-operated mass lesion (Marshall 6 category); b) GCS = 3 and bilateral, fixed and dilated pupils; c) an unclear history of the mechanism of injury as the primary event; d) hypotension, that is, systolic blood pressure persistently < 90 mmHg during the observation period; e) the need for cardiopulmonary resuscitation; f) penetrating injuries at presentation; and g) discharge against medical advice.

The use of antiplatelet drugs was systematically recorded, independent of time of exposure. Aspirin (usual dose, 100 mg), ticlopidine, indobufen (a popular antithrombotic drug used in Italy) and clopidogrel were considered, as well as the potential antiplatelet activity of other anti-inflammatory agents. During the observation period there was no specific indication for rescue therapy with human prothrombin complex or platelet transfusions in subjects treated with anticoagulant/antiplatelet agents, and no patients received this support treatment.

### Treatment protocol

From the ED, subjects were transferred for observation and treatment to a high dependency unit, ordinary admitting unit, neurosurgical unit or ICUs. After admission, all patients were submitted to additional head CT scan within 6 to 24 hours from injury according to local protocols. Furthermore, CT was always repeated in the case of clinical or neurological deterioration. The time interval between trauma and the initial head CT scan was dictated by emergency procedures of the individual centers. For the purpose of the present study, all head CT scans were retrospectively reviewed in a temporal sequence by an independent expert neuroradiologist in a blinded fashion to confirm the initial diagnosis and to evaluate possible worsening in the head CT scan at 6 to 24 hours. CT scans were classified according to the criteria of Marshall [10], modified according to the revision of the European Brain Injury Consortium (EBIC) [11].

The protocol was carried out according to the Helsinki Declaration and approved by the ethical committee of the Local Health District of Forlì. All data were transferred from the peripheral centers to the coordinating unit in a completely anonymous form. According to the Italian law on privacy protection and use of personal data (Dls n. 85, March 1, 2012), informed consent is not needed whenever handling is carried out in an anonymous form on retrospective data on file and it would be technically impossible to trace people for signing consent forms.

### Variables definition

A few items were selected as the variables potentially associated with outcomes. We considered age, sex, type of injury (motor vehicle accidents, falls or accidental, work-related, assault, sport injuries and other causes), coagulation (by prothrombin time) and neurological status (by GCS), as well as the use of antithrombotic agents as described above. In the antiplatelet group, we also considered the few cases in which other non-steroidal anti-inflammatory drugs (NSAIDs) with a definite antiplatelet activity had been administered in the three days before trauma for other reasons.

Comorbidities, although common and associated with outcome in spontaneous intracerebral hemorrhage [8,9], were not considered in the present analysis. In previous studies on traumatic brain injury comorbidities did not predict short- or long-term outcome [1].

The intracranial injuries considered for analyses were: traumatic subarachnoid hemorrhage (t-SAH), subdural hematoma (SDH), epidural hematoma (EDH), intracerebral hemorrhage/contusion (ICH) or depressed skull fracture (DSF) and intraventricular hemorrhage (IVH) [12,13]. IVH was considered a distinctive intracranial injury, but no subjects were considered with positive head CT scans for this type of injury as a unique lesion. In all cases IVH resulted in different combinations with other types of intracranial injury.

Patients' coagulation status (prothrombin time) was determined by protocol in all cases. Values of the International Normalized Ratio (INR) > 1.5 were considered at risk of hemorrhage.

### Outcome measures

Short-term outcome measures were: a) intracerebral injuries with worsening characteristics, indicated by a change of at least one point in Marshall category between initial and follow-up CT scan performed during serial controls within 24 hours; and b) the need for neurosurgical intervention because of clinical and/or radiological deterioration during the observation period. This period was limited to the first seven days after diagnosis in order to exclude delayed complication of injury (chronic subdural hematomas, hygromas or hydrocephalus) [12].

As a long-term outcome measure we considered the Glasgow outcome scale (GOS) at six months. For ease of analysis and reporting, the five-point GOS score was categorized as either favorable (moderate disability or good recovery - GOS 4 to 5) or unfavorable (dead, vegetative, or severely disabled - GOS 1 to 3). The follow-up GOS was rated by an expert physician unaware of the study protocol, on the basis of the response to a structured telephone call [12]. Main outcome measures were then related to the different hospital facilities, for example, hospital with neurosurgical unit, hospital with telemedicine consultation only (no neurosurgical unit) and hospital without both neurosurgical unit and telemedicine consultation.

### Statistical analysis

A data mining method was chosen to select relevant patterns between predictor variables and main outcomes by Weka software (University of Waikato, Hamilton, NZ). We used a decision tree technique, in which nodes indicate decision points, chance events, or branch terminals. Branches correspond to each decision alternative or event outcome emerging from a node. The root nodes are the first set of decision alternativeness. The construction of a decision tree was obtained by a 'recursive partitioning' analysis [14].

Mean value, SD and frequencies were used to describe data distribution. We used multivariable logistic regression analysis with a *P *value greater than 0.05 for removal of variables. A score for the risk of unfavorable outcome was calculated for each patient on the basis of the coefficients computed by the logistic regression derived from variables entering the stepwise procedure. The accuracy of the risk score was then evaluated by the area under the receiver operating characteristic (ROC) curves. The odds ratio (OR) and 95% CI were also calculated. We tested the association of each variable with the primary outcome measure using Chi-square tests for nominal variables, the Mann-Whitney U test for ordinal variables, and the unpaired two-tailed *t*-test for continuous variables (SPSS software, version 17.0 - SPSS Inc., Chicago, IL, USA). The relative risk (RR) of different outcomes was also calculated.

## Results

### Patients

The mean age of the 1,558 subjects with intracranial lesions was 65 years (SD 21), with 288 (18.5%) patients under 40 and 664 subjects (42.6%) over 75. The vast majority of subjects (1,123 cases, 72.1%) had a mild head injury with GCS 14 to 15, 420 cases (24.9%) had a moderate injury (360 cases with GCS 13 to 11 and 60 with GCS 10 to 9). The last group of 15 subjects (1.0%) had a Marshall category 2 to 4 and severe head injury (GCS < 9) (Table 1).

**Table 1:** Clinical characteristics of subjects according to worsening characteristics between initial and follow-up CT scan

	Worsening (Number = 201)	Stable/Improved (Number = 1.357)	OR (95% CI)	*P *value
Sex (males)	125 (62.2%)	786 (57.9%)	1.19 (0.88 to1.62)	0.283
Age (mean: SD)	65 (22)	65 (21)	--	--
Mechanism of Injury				
Road accident	60 (29.9%)	414 (30.5%)	0.97 (0.70 to 1.34)	0.870
Other causes	86 (42.8%)	622 (45.8%)	Reference	--
Glasgow Coma Scale				
Moderate-Severe (≤ 13)	127 (63.2%)	308 (22.7%)	5.84 (4.27 to 8.00)	< 0.001
Mild (15 to 14)	74 (36.8%)	1.049 (77.3%)	Reference	--
Basal skull fracture	28 (13.9%)	117 (8.6%)	1.71 (1.10 to 2.67)	0.019
Type of lesion				
Subdural hematoma	106 (52.7%)	498 (36.7%)	1.92 (1.43 to 2.59)	< 0.001
Epidural hematoma	28 (13.9%)	129 (9.5%)	1.54 (0.99 to 2.39)	0.059
Intracerebral hemorrhage/contusion	116 (57.7%)	650 (47.9%)	1.48 (1.10 to 2.0)	0.010
Traumatic subarachnoid hemorrhage	105 (52.2%)	671 (49.4%)	1.12 (0.83 to 1.50)	0.497
Intraventricular hemorrhage	10 (4.9%)	84 (6.2%)	0.79 (0.40 to 1.55)	0.634
Depressed skull fracture	25 (12.4%)	116 (8.5%)	1.52 (0.96 to 2.41)	0.086
Anticoagulant therapy	18 (9.0%)	108 (8.0%)	1.14 (0.68 to 1.92)	0.582
Antiplatelet therapy	106 (52.7%)	431 (31.8%)	2.40 (1.78 to 3.23)	< 0.001

Data are reported as number of cases and %, or median and standard deviation; SD.

A total of 708 subjects (45.4%) were injured by falls or accidents with 474 (30.4%) following a road accident. In the remaining subjects the head injury was work-related (83 cases, 5.3%) or following an assault (46, 3.0%), or related to sports and other causes (247, 15.8%) (Table 1).

At the first evaluation, 1,328 subjects (85.2%) had an intracranial injury with Marshall category 2, 168 subjects (10.8%) had category 3, and only 62 cases (4.0%) had category 4 (Table 1). A single lesion was recorded in 886 subjects (56.9%), 2 lesions in 430 cases (27.6%) and 3 or more lesions in the remaining 237 cases (15.2%). The frequency distribution of type of lesion was: ICH (766 cases; 49.2%), SDH (604; 38.8%), t-SAH (776; 49.8%), EDH (157; 10.1%) and IVH (94; 6.0%) (Table 1).

Pre-injury antiplatelet therapy was recorded in 537 subjects (34.5%) of the entire cohort (454, 49.1% in the group ≥ 65 years old). Aspirin was the most frequently used antiplatelet medication (439 subjects, 28.2%), followed by ticlopidine (69, 4.4%), clopidogrel (28, 1.8%), NSAIDs (20, 1.3%) and low molecular weight heparin (10, 0.6%). A group of 129 cases (8.3%) had INR > 1.5 because of simultaneous treatment with warfarin.

### Outcome prediction

#### Short-term outcome

**Figure 1:**
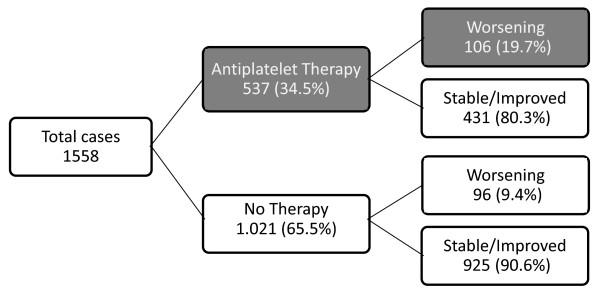
**Distribution of worsening events in relation to antiplatelet therapy in subjects with intracranial lesions following head injury**. Significant outcomes in the decision tree analysis are reported as white text on a grey background.

In 201/1,558 subjects (12.9%) head CT scan comparison documented a worsening lesion in the short-term. Antiplatelet therapy increased the risk of worsening two-fold (*n *= 106, 19.7% of treated versus 95, 9.4% of untreated cases), corresponding to a relative risk (RR) of 2.09, 95% CI 1.63 to 2.71 (Figure 1). Compared with untreated subjects, the risk was particularly high in subjects on clopidogrel (RR 5.76, 95% CI 3.88 to 8.54), independent of the association with aspirin (15 cases, 8 with worsening lesions; RR 5.73, 95% CI 3.44 to 9.55; *P *< 0.001).

On multivariable logistic regression analysis a group of 5/14 items was independently associated with worsening lesion (Table 2). The discriminating operating characteristics area of the selected items was 0.777 (95% CI 0.755 to 0.797; *P *< 0.001).

**Table 2:** Logistic model of variables considered in predicting subjects with worsening lesions after head injury

Covariates	Odds Ratio	95% CI	*P *value
Sex (males)	1.24	0.88 to 1.75	0.211
Age (decades)	0.91	0.83 to 1.01	0.065
Road accidents	1.03	0.70 to 1.52	0.874
Glasgow Coma Scale	4.59	3.23 to 6.51	< 0.001
Basal skull fracture	1.20	0.72 to 1.99	0.480
Marshall category	1.43	1.09 to- 1.89	0.011
Type of lesion			
Subdural hematoma	1.32	0.86 to 2.01	0.205
Epidural hematoma	1.13	0.65 to 1.98	0.656
Intracerebral hemorrhage/contusion	0.96	0.62 to 1.50	0.875
Traumatic subarachnoid hemorrhage	0.80	0.51 to 1.24	0.322
Intraventricular hemorrhage	0.37	0.17 to0.775	0.008
Depressed skull fracture	1.03	0.60 to 1.78	0.903
Number of lesions (≥ 2)	2.56	1.46 to 4.51	0.001
Anticoagulant therapy	1.17	0.65 to 2.10	0.606
Antiplatelet therapy	2.87	1.94 to 4.23	< 0.001

Marshall category (category 2-3-4) and age (decades) were considered as continuous variables, Glasgow Coma scale for categories (mild and moderate-severe; the lower score the higher risk). The remaining variables were dichotomized.

**Figure 2:**
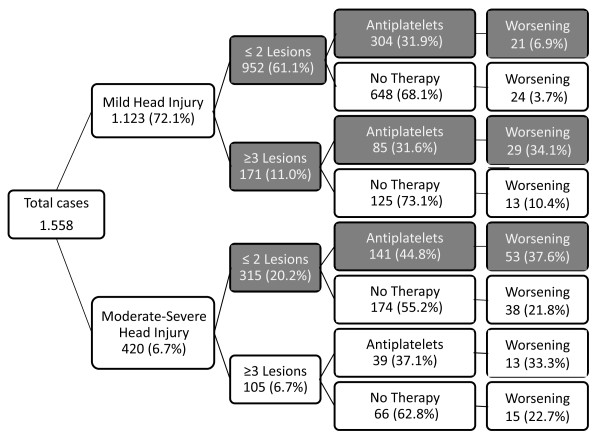
**Data mining analysis: relevant patterns of variables predicting cases with worsening lesions in relation to severity of head injury (mild versus moderate-severe head injury), number of intracranial lesions and antiplatelet therapy**. Significant variables are reported as white text on a grey background.

Data mining analysis selected the following relevant patterns between predictor variables and main outcomes: a) in subjects with mild head injury (GCS 15 to 14), antiplatelet therapy increased the risk of worsening two-fold when the number of lesions at the first CT scan was ≤ 2, (6.90% treated versus 3.70% not treated; RR 1.86, 95% CI 1.06 to 3.30, *P *= 0.032) and further increased the risk of worsening when the number of lesions was ≥ 3 (34.8% treated versus 10.4% not treated; RR 3.34, 95% CI 1.74 to 6.40, *P *= 0.003) (Figure 2); b) in subjects with moderate-severe head injury antiplatelet therapy increased the risk of worsening when the number of lesions at the first CT scan was ≤ 2 (37.6% treated versus 21.8% not treated, RR 1.72, 95% CI 1.21 to 2.45; *P *= 0.002 (Figure 2).

Worsening seen on serial CT scans resulted in neurosurgical intervention in 46 subjects (2.9%). The intervention was required for EDH (8 cases), SDH (30 cases) and ICH (8 cases). Neurosurgical intervention was needed more frequently in subjects treated with antiplatelet drugs (21.2% treated versus 11.2% untreated, RR 1.90, 95% CI 1.35 to 2.66; *P *< 0.001). On multivariable logistic regression analysis 8/15 items (male sex, younger age, mechanism of injury, INR > 1.5, antiplatelet therapy, GCS, Marshall category, and type of lesions) were independently associated with worsening and the need for neurosurgical intervention.

#### Long-term outcome

A complete six-month follow-up was obtained in 1,222/1,558 subjects (78.4%). A total of 336 cases (21.6%) were lost at follow up and in 115 (7.4%) cases GOS was unreliable due to previous disability or trauma-related disability not dependent on head injury.

**Figure 3:**
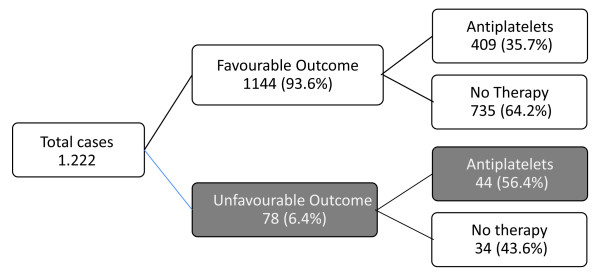
**Unfavorable outcomes in subjects with head injury and intracranial lesions in relation to antiplatelet therapy**. Significant outcomes are reported as white text on a grey background.

Outcome was unfavorable in 78 cases (5.0%): 26 patients (1.7%) died during the six-month follow up, 9 patients (0.6%) were judged in a permanent vegetative state and 43 (2.8%) were severely disabled. The majority of subjects (*n *= 1,144, 73.4%) had a favorable outcome, with moderate disability being present in only 168 cases (10.8%). At follow up, the risk of unfavorable outcome at six months increased by 50% in the group treated with antiplatelet therapy (9.7% treated versus 4.4% untreated; RR 1.58, 95% CI 1.28 to 1.95; *P *< 0.001) (Figure 3).

On multivariable logistic regression analysis only 4/14 items (GCS, Marshall severity, age in decades, intracerebral hemorrhage/contusion) were selected as predictors of unfavorable outcome (Table 3). The discriminating operating characteristics area of the selected items was 0.891 (95% CI 0.860 to 0.921); *P *< 0.001.

**Table 3:** Logistic model of variables considered in predicting unfavorable outcome in subjects with head injury

Covariates	Odds Ratio	95% CI	*P *value
Sex (males)	1.30	0.75 to 2.26	0.348
Age (decades)	1.33	1.11 to 1.59	0.002
Road accidents	0.85	0.42 to 1.70	0.641
Glasgow Coma Scale	12.94	6.26 to 26.78	< 0.001
Basal skull fracture	0.87	0.35 to 2.12	0.754
Marshall category	3.03	2.09 to 4.39	< 0.001
Type of lesion			
Subdural hematoma	0.57	2.78 to 1.16	0.119
Epidural hematoma	0.78	0.31 to 2.00	0.607
Intracerebral hemorrhage/contusion	0.46	0.22 to 0.94	0.034
Traumatic subarachnoid hemorrhage	0.76	0.36 to 1.62	0.481
Intraventricular hemorrhage	2.07	0.86 to 4.96	0.104
Depressed skull fracture	0.75	0.25 to 2.24	0.608
Number of lesions (≥ 2)	1.99	0.77 to 5.10	0.153
Anticoagulant therapy	1.01	0.44 to 2.32	0.971
Antiplatelet therapy	1.02	0.57 to 1.84	0.938

Marshall category (category 2-3-4) and age (decades) were considered as continuous variables, Glasgow Coma scale for categories (mild and moderate-severe; the lower score the higher risk). The remaining variables were dichotomized.

These results were confirmed by ordinal regression analysis: five items (Marshall severity, GCS, age in decades, antiplatelet therapy and type of injury) were selected for the prediction of GOS with a discriminating operating characteristics area of 0.716 (95% CI 0.645 to 0.786; *P *< 0.001).

These results were not significantly different after exclusion of subjects treated with warfarin or low molecular weight heparin (LMWH) (discriminating operating characteristics area of 0.783 (95% CI 0.746 to 0.820; *P *< 0.001)) and in the subgroup of subjects fully recovered or with moderate disability at six months, that is, GOS 4 to 5 (0.716, 95% CI 0.645 to 0.786; *P *< 0.001).

Data mining analysis did not select any relevant pattern in relation to different hospital facilities, that is, neurosurgery versus telemedicine systems versus none; *P *test for trend = 0.144).

## Discussion

This observational study derived from Italian EDs shows that pre-injury antithrombotic therapy is associated with negative outcomes in subjects with head injury and intracranial lesions with an indication of observation and conservative treatment; in the short-term progression of lesions was seen on the CT scan, in the long-term the risk of unfavorable outcome increased. The risk of lesion worsening was particularly high when subjects were treated with clopidogrel, independent of the concomitant use of other antiplatelet agents.

The prognosis of subjects with head injury and intracranial lesions with an indication for conservative treatment is extremely variable, depending on the progression of injury, the size of the lesion and secondary injury responses that may worsen the primary lesion [15]. The earlier the initial CT scan, the greater the likelihood that the lesions will progress at follow-up. Progression generally occurs within the first 12 hours, but may occur as late as three to four days after trauma. Small contusions that progress are usually clinically silent and are less likely to require neurosurgical intervention [16], whereas large contusions in subjects with low GCS scores are more likely to evolve [15].

Injury progression was defined by worsening of the Marshall category, a validated tool to assess the outcome of subjects with head injury [17-19]. According to EBIC [11], an increase in the Marshall CT category at the follow-up CT scan may be considered a sign of disease progression. Whenever the initial CT scan shows a diffuse injury without swelling or shift worsening to a mass lesion with need of neurosurgical intervention, the outcome becomes definitely unfavorable (62% versus 38%) [20].

Our data confirmed that the risk of imaging progression is associated with the severity of the initial Marshall category with 10.2% of cases worsening in the group of subjects classified as Marshall category 2, 25.6% of subjects in category 3 and 37.1% of subjects categorized as Marshall 4. Our worsening rate is, however, much lower than that reported in different series from neurosurgical facilities, where approximately 50% of patients with lesions who were admitted for conservative treatment showed progression [21-24]. This difference is probably due to the selection of more severe and younger patients in neurosurgical units, including Marshall 5 cases, compared to those observed in a general ED. This hypothesis is also confirmed by a larger use of neurosurgical evacuation reported in those settings, whereas neurosurgery may be contraindicated in older persons, although this issue is not settled.

A group of variables (injury severity, anticoagulant therapy, need for cardiopulmonary resuscitation in the field, older age, short duration between injury and the first CT scan, multiple lesions, midline shift and injuries with need of neurosurgical procedures) had been indicated as predictors of radiological progression [22,23,25,26]. Our study confirms the importance of clinical and radiological items selected by previous studies in predicting lesions likely to evolve after head injury and indicates antiplatelet therapy as a relevant, additional predictor.

The negative effect of antiplatelet therapy might depend on a number of factors, such as minimum continued bleeding or a microvascular dysfuntion, exaggerated by reduced platelet function, favoring edema and brain swelling, producing a midline shift [15].

We defined progression on the basis of the Marshall classification. Differences in radiological progression may depend on the criteria used: 100% [26], 30% [21] or 25% increase [22] in hematoma dimension, but different cuts and angulation may introduce an important bias with the use of strict criteria, such as a 25% to 30% enlargement [11]. The Marshall category, although crude, provides a very easy-to-define clinical index of progression. It was selected as an outcome measure in the short term and, combined with GCS, it was the only variable associated with unfavorable outcome at six months, confirming the clinical importance of this item.

Among antiplatelet agents both aspirin and, particularly, clopidogrel increased the risk of evolving lesions, but their combined use did not further increase the risk. By contrast, ticlopidine, largely used in Italy in the past, did not increase the risk. The risk associated with clopidogrel is of particular concern, considering its increasing use. The advantages of clopidogrel on cardiovascular outcomes have made it a lifesaving drug in subjects over 45 years old who have cardiovascular disease [5,27]. Its use was later extended from coronary artery disease to cerebrovascular and peripheral artery disease, thus being largely diffused in the elderly population [28]. An analysis of drug prescriptions in more than 300,000 Italian subjects with diabetes showed an increased prevalence of antiplatelet drug use from 15% to 52% in the period 1997 to 2006 [29], and in 2008 more than 4% of the general Italian population was treated with aspirin [30]. In our series, approximately 35% of the subjects were being treated with antiplatelet agents, and this figure increased to 53% in the group of subjects more than 75 years old. The use of these drugs is likely to increase further in the future, following guidelines indicating antiplatelet drug therapy in a large proportion of older subjects [5,27].

Two recent reviews have summarized the available evidence on the risk of unfavorable outcomes of antiplatelet medications, especially in subjects with severe head injury and older age. Beynon *et al. *[31], on the basis of the scarce available evidence, concluded that these agents increase the risk of an unfavorable outcome, particularly in cases of severe traumatic brain injury. In a meta-analysis of five studies, Batchelor and Grayson compared the mortality rates of patients with blunt head trauma who were on aspirin or clopidogrel versus cases not on antiplatelet agents [32]. They found a significant heterogeneity and a moderately increased overall risk of death for both drugs, which did not reach statistical significance. However, the low number of events precludes any firm conclusion and further work is required.

Event rates constitute an even more significant drawback in studies on mild-to-moderate brain injury. Nonetheless, the mortality rate of subjects receiving aspirin was also reported to be higher than normal [33]. In cases observed in EDs, pre-injury antiplatelet therapy was recently shown to increase significantly the risk of intracranial lesions in subjects after mild head injury [34], whereas in a prospective study of mild and moderate head injury in subjects more than 60 years old, low-dose aspirin prophylaxis had no effect on the frequency or types of intracerebral or meningeal hemorrhage [35]. The initial size of the contusion and the presence of SDH were selected as predictors of radiological progression, and the initial GCS and younger age as predictors of good disposition at discharge [34], but much more evidence is required before a firm conclusion can be drawn. Anticoagulation and antiplatelet therapy were not included in any study model [21].

Age represents an important issue in head injured patients. In Italy, age is not formally considered a criterion for admitting patients to hospitals with different levels of care, but in clinical practice older patients frequently have limited access to conservative observation in neurosurgical units and to interventions. Our analysis selected older age as a significant, independent predictor of long-term outcome. In the Italian database, 924 subjects (59.3%) were ≥ 65 years old and 42% were older than 75, a figure completely different from previously published studies. In a widely cited study [36], subjects older than 65 years were excluded from the analyses, the median age of subjects was 33 when treated in neurosurgical units and 31 in those admitted to non-neurosurgical centers. Our database reflects the 'real world' of head injury subjects with Marshall 2 to 4, observed in the Italian EDs, with a median age of 72 years and with 30% of the cases older than 80, as previously reported [37,38].

The growing elderly population and the expanding indications for anticoagulant therapy might produce more complications associated with anticoagulant treatment, challenging the emergency physicians more and more. A very recent study showed that oral anticoagulants may also be safely used in older patients at risk of fall [39], but in a previous report we showed that anticoagulation increased the risk of intracranial lesions by more than four times, independent of other variables [12]. In the present study, anticoagulant treatment did not significantly predict worsening in the 126 cases (8.1%) on anticoagulants with an INR above 1.5, but a selection bias may be operative. In subjects on oral anticoagulants, the initial lesion might be so severe (that is, Marshall 5 or 6) that is excludes them from the analysis. The progressive use of rapid anticoagulation reversal will clarify this problem.

A few limits should be considered. Firstly, selection biases might be present because of the retrospective nature of the analysis of clinical records and different extraction procedures according to software available in the various EDs. These biases might be amplified by incomplete recording of drug use and/or incomplete reporting by patients. Underreporting of drug use might also increase in relation to incomplete anamnesis by physicians, unaware of the possible risk associated with antiplatelet drugs.

Secondly, the time lag between head trauma and CT scanning was variable between a few hours to 24 hours. Both trauma-to-admission and admission-to-CT times were variable, according to clinical judgment, with an influence on the natural history of lesions. As discussed above, these biases might be reduced by the use of the Marshall classification, in which category changes imply evident changes in the imaging appearance of lesions.

Thirdly, the history of antiplatelet drug use might be completed by the analysis of antiplatelet activity. In a series of 84 subjects treated with aspirin, 2.4% of cases had normal platelet function, and 42% of subjects without a documented history of aspirin use had platelet inhibition. Aspirin resistance is a multifactorial phenomenon, associated with comorbidities, leading to reduced platelet activation and aggregation [40,41]. However, aspirin history and the measured activity of platelet inhibition were associated with only a marginal risk of CT scan progression, craniotomy, mortality or poor outcome at multivariable analysis [42].

Fourthly, we did not consider comorbidities in our analysis. Comorbidities have a definite importance in hemorrhagic stroke [8] and spontaneous intracerebral hemorrhage [9], whereas their importance in traumatic lesions is doubtful. The use of antiplatelet drugs might identify subjects with more prevalent cardiovascular disease, at higher risk of spontaneous cerebro-vascular events, independent of antiplatelet use. Apparently, this does not apply to traumatic brain lesions and the Charlson index of comorbidities was not associated with outcome in a previous study in Italian EDs [1,2].

Finally, an increase in the Marshall CT classification score would not always represent lesion extension or disease progression. The hierarchy of Marshall class is based on the absence/presence of signs of raised intracranial pressure, such as brain swelling, midline shift and mass lesions which need neurosurgiucal evacuation. This is very likely to determine an unfavorable outcome in the long term, but this is not always the case. Lack of significance of variables other than Marshall category and GCS versus outcome determined by GOS categories does not exclude possible clinical relevance.

## Conclusions

Our data, which are derived from a representative number of Italian EDs, show that pre-injury antithrombotic therapy is associated with an increased risk of short-term radiological worsening and six-month unfavorable outcome in subjects with a positive head CT scan, particularly in subjects treated by clopidogrel. The results should be considered in predictive algorithms of future guidelines of diagnosis and treatment of head injury.

## Key messages

• In subjects with mild or moderate-severe head injury and a positive head CT scan with indications for conservative treatment, 12.9% of subjects worsened by CT comparison (change of at least one point in the Marshall category) at 6 to 24 hours.

• A group of 5/14 items (GOS, Marshall category, antiplatelet therapy, IVH, number of lesions) were independently associated with short term (6 to 24 hours) worsening.

• Pre-injury antiplatelet therapy increased the risk of short term worsening two-fold. The risk was particularly high in subjects on clopidogrel, independen of the association with other antiplatelet drugs.

• At long-term follow up (six months), only 4/14 items (GCS, Marshall severity, age in decades, intracerebral hemorrhage/contusion) were selected as predictors of unfavorable outcome. The risk increased by 50% in the group treated with antiplatelet therapy.

## Abbreviations

CT: computed tomography; DSF: depressed skull fracture; EBIC: European Brain Injury Consortium; EDs: emergency departments; EDH: epidural hematoma; GCS: Glasgow coma scale; GOS: Glasgow outcome scale; ICH: intracerebral hemorrhage/contusion; INR: International Normalized Ratio; IVH: intraventricular hemorrhage; NSAIDs: non-steroidal anti-inflammatory drugs; RR: relative risk; SDH: subdural hematoma; SIMEU: Società Italiana di Medicina d'Emergenza-Urgenza; t-SAH: traumatic subarachnoid hemorrhage.

## Competing interests

The authors declare that they have no competing interests.

## Authors' contributions

AF conceived the study, wrote the protocol, coordinated data collection and the interpretation of results and wrote the paper. FS and CB contributed to interpretation of the results and critical review of the paper. GM contributed to study design, interpretation of the results and co-wrote the paper. DM and LA contributed to statistical analyses and data mining, and the S.I.M.E.U. Study Group for data collection. All authors read and approved the final version of the paper.
